# Teachers’ Mental Health and Their Involvement in Educational Inclusion

**DOI:** 10.3390/bs12080261

**Published:** 2022-07-29

**Authors:** Silvia Salinas-Falquez, Carlos Roman-Lorente, Mirela Buzica, Joaquín Álvarez, Nieves Gutiérrez, Rubén Trigueros

**Affiliations:** 1 Department of Psychology, University of Guayaquil, Guayaquil 090514, Ecuador; silvia.salinasf@ug.edu.ec; 2 Department of Psychology, Hum-878 Research Team, Health Research Centre, University of Almeria, 04120 Almeria, Spain; carlosromanlorente@yahoo.es (C.R.-L.); mirelaileana1979@gmail.com (M.B.); jalvarez@ual.es (J.Á.); nga212@ual.es (N.G.)

**Keywords:** frustration of basic psychological needs, Index for Inclusion, resilience, emotional intelligence

## Abstract

Teaching is one of the most stressful work contexts, psychologically affecting professionals. The objective of this study is to analyse the effect of the frustration of NPB basic psychological needs, resilience, emotional intelligence and inclusion from the perspective of teachers in the time of the COVID-19 pandemic. The study is carried out with 542 teachers of therapeutic pedagogy and special educational needs using the Psychological Need Thwarting Scale PNTS questionnaires as a research method, the Resilience Scale (RS-14), the Trait Meta Mood Scale 24 (TMMS-24), the Maslach Burnout Inventory, and the Index for Inclusion. The results revealed positive correlations, on the one hand, between the factors of frustration among themselves and with burnout and, on the other hand, the positive correlation between emotional intelligence, resilience and the inclusion index. In conclusion, the resilience of teachers plays a protective role in the inclusion of students with SEN in the face of emotional exhaustion and the frustration of psychological needs.

## 1. Introduction

Teaching is one of the most stressful work contexts, with a great tendency for professionals to be psychologically affected [1]. In this sense, it is a work environment which, due to its conditions, requires teachers to face complex situations with high emotional involvement [2]. This demand is marked by workload, lack of social and institutional support, and classroom management difficulties [3,4]. To the stress already experienced by teachers, a new factor was added, the global COVID-19 pandemic, after which all teaching staff had to adapt quickly to provide access to online educational materials for students and to undertake measures to prevent the spread of the disease in the classroom. Given this situation, teachers have reported high levels of stress and burnout [5] leading to burnout syndrome, which is characterised by a set of symptoms and signs as a response to chronic stress [6], with high consequences on the health and psychological and physical well-being of the individual [7], and effects such as job abandonment, increased absenteeism, and deterioration of the service offered [6]. Among these symptoms we can highlight physical symptoms, such as headache, myalgia, and hypertension [8]; behavioural symptoms, such as attention deficit, aggressiveness, inflexibility, rigidity, inability to relate to others, and isolation; emotional symptoms [9], such as irritability, anxiety [10], disorientation, impatience, and hostility; and cognitive symptoms, such as low self-esteem, low performance at work, professional failure, etc. Consequently, such burnout hinders the achievement of objectives, diminishing teachers’ feelings of self-efficacy and, over time, giving rise to burnout syndrome [11,12]. However, despite the negative psychological consequences of the excessive workload to which teachers are subjected and the consequences that COVID-19 generates and has generated, there are a series of internal psychological mechanisms that facilitate the adaptation and overcoming of the individual to the possible vicissitudes that arise. According to Bisquerra [13], a key role in school coexistence is played by the EI level of the teacher and his or her ability to control emotions in the classroom [13]. In this sense, resilience and emotional intelligence are two of the most important mechanisms that could help teachers to cope positively with daily stressors, facilitating their work to meet the special educational needs of the students they serve. Thus, the present study aims to analyse the psychological coping mechanisms of teachers when facing burnout symptoms arising from daily challenges, and the consequences this has on their inclusive behaviours.

### 1.1. Frustration of Basic Psychological Needs

According to self-determination theory [14] human behaviour is motivated by three basic psychological needs (BPN): autonomy, competence and relatedness to others. Autonomy refers to the need for each person to feel that he/she is able to regulate and control his/her behaviour [15]; competence relates to the person’s involvement in different situations that challenge his/her abilities and are presented as challenging situations for him/herself [16]; finally, relatedness is linked to people’s need for interaction and a sense of belonging [17]. These three needs are essential to facilitate the optimal functioning of natural tendencies for growth and integration, as well as for social development and personal well-being. NPBs can be frustrated or satisfied. The frustration of needs predicts burnout while satisfaction is highly correlated with vitality and personal development [18]. When NPBs are hindered it implies a change in maladaptive or even maladaptive motivational functioning; however, when they are satisfied, positive effects are produced regardless of people’s goals, interests, values and preferences [18], i.e., better performances are promoted.

In terms of previous research, the study by Bartholomew et al. [19] shows that the frustration of NPB increases demotivation, emotional exhaustion and cynicism in teachers. On the other hand, they highlight that those teachers who are more frustrated with their competence are those who have less intrinsic motivation and are more demotivated at work. Likewise, Bartholomew et al. [20] found that frustration with autonomy, competence and relationships with others increases burnout. In the same vein, Van den Berghe et al. [21] state that NPB satisfaction increases the negative factors of burnout and significantly reduces professional effectiveness.

### 1.2. Emotional Intelligence

Emotional intelligence is defined as the ability to know and manage one’s own and others’ emotions, to feel satisfaction and to be effective in life [22]. According to Bar-On [23], social–emotional intelligence is a cross-section of interrelated emotional and social competencies, skills and enablers that determine how effectively we understand and express ourselves, understand and relate to others, and cope with daily demands. This model is based on the individual, on the ability to understand one’s own weaknesses and strengths, and the ability to express one’s feelings and thoughts in a non-destructive way. In this sense, EI influences adaptation processes, facilitating appropriate responses to the different events that a person has to face in their daily life, reducing maladaptive emotional reactions, enabling the experience of positive moods, and reducing the incidence of negative ones [24].

Goleman [22] states that emotionally intelligent people possess a series of characteristics, such as: self-awareness, self-regulation, empathy, social skills, and self-motivation. All this helps a person to know themselves and to know and understand the moods they have; they know how to control impulses and emotions, and are able to think before acting, that is, they are assertive, open to new ideas and have the capacity for flexible thinking in the face of change; they are able to put themselves in the place of others and not just listen to them; they know how to manage their social skills to relate to all kinds of people; and they are able to motivate themselves without expecting to be recognised or receive a prize for their achievements, with this strength and motivation coming from within.

Several studies have provided evidence of the positive relationship between EI and psychological adjustment [25], well-being [26], social functioning [27], and health [28]. Along these lines, a growing body of research reports that teachers’ personal competences, and more specifically, EI, are extremely important for their professional performance [29,30]. Teachers with high levels of EI have been found to be less vulnerable when facing stressful work situations, as they feel skilled in regulating their emotions, are better able to develop active strategies to cope with stressful situations in the academic environment, and enjoy greater personal fulfilment and less stress [31,32].

### 1.3. Resilience

Resilience comprises two levels: the first is resistance or the ability to cope with the “problem” and the second is the ability to “build” or rebuild positively in spite of difficulties [33]. Resilience is a positive attitude towards life despite difficult circumstances and represents the positive side of mental health. It also consists of knowing how to learn from defeat and transform it into an opportunity for personal development. In this sense, Vanistendael and Saavedra [33] distinguishes two components: on the one hand, resistance to destruction, i.e., the ability to protect one’s own integrity under pressure and, on the other hand, the ability to forge a positive life behaviour despite the difficult circumstances that the subject is going through, i.e., resilience can be improved and trained to deal positively with adversity.

The most resilient people maintain a greater emotional balance in stressful situations, which allows them to better withstand pressure and, consequently, experience a greater sense of control and ability to cope with difficult situations [12]. A resilient person is not an exceptional being: they can be anyone, i.e., resilience is in the person and in the variables of their immediate environment. In other words, resilience is created by the person’s temperament, culture, cultural meaning and social support. It could be said that the socio-cultural context in which the individual lives can favour or hinder the development of resilience, train them, and improve the person’s capacities, transforming him/her into a resilient person.

According to Vicente de Vera and Gabari [34], resilience in secondary school teachers could be a modulating variable of teacher distress, making it easier for teachers to adapt to or overcome stressful situations and, consequently, would lead to greater dedication and motivation to meet their objectives and therefore respond adequately to the demands of the profession [34]. Likewise, the study carried out by Mérida et al. [35] concluded that resilience and emotional intelligence have a positive influence on the behaviour of teachers, increasing their commitment to their teaching work. In addition, it has been found that the use of resilient coping strategies and teacher training in this area could reduce the deterioration of stress produced by the arduous work of teaching [36]. Along the same lines, the study by Díaz and Barra [37] concluded that the dispositional characteristics of resilience would be protective factors that would allow teachers to be satisfied with their teaching work despite the difficult scenario in which they carry out their role. According to Zadok-Gurman et al. [38], in difficult times, such as a pandemic, resilience can reduce the adverse effects of stressors on mental health and promote positive mental health [38]. Finally, the work of Garcia [39] and Vicente de Vera and Gabari [40] conclude that resilience decreases vulnerability to burnout.

### 1.4. Burnout

The term burnout is understood as a gradual process by which people gradually lose interest in their work and responsibilities, and can lead to deep depression. Maslach and Jackson [41] define burnout as a behavioural manifestation of work-related stress, and understand it as a three-dimensional syndrome. This three-dimensional construct is characterised by three main manifestations: emotional exhaustion, depersonalisation, and low self-fulfilment [42]. One of the characteristics of burnout syndrome is exhaustion, causing the person to feel overwhelmed and tired by the performance of their work, causing a decrease in interest and job satisfaction [43]. In the same vein, Cortez-Silva et al. [44] say that burnout syndrome is a response to chronic emotional and interpersonal stressors at work, and emotional exhaustion is one of the components of burnout syndrome.

### 1.5. Teachers and COVID-19 Literature Review

The COVID-19 pandemic has further challenged teachers, increasing emotional exhaustion and the deterioration of their competences, resulting in a loss of sensitivity and empathy and a high sense of failure Cortez-Silva et al. [44]. This leads to decreased job and personal performance and job dissatisfaction [45]. A study by Cevallos et al. [46] and Duan and Zhu [47] concluded that the adaptive process to which teachers were subjected during the COVID-19 pandemic has caused them high physical and psychological exhaustion, highlighting, on the one hand, that symptoms of anxiety, depression and stress were the most common reactions among teachers. On the other hand, a study by Kukreti et al. [48] showed that teachers’ perceived fear of COVID-19 led to an increase in psychological stress and post-traumatic stress, resulting in an increase in absenteeism.

A study by Eşici et al. [49] showed that teachers have a need for psychological support and continuous training due to the problems experienced in adapting to the new teaching situation, especially regarding pupils’ access to education. Similarly, a study by Sugianto and Ulfah [50] showed that the pandemic led to an increase in teacher insecurity, anxiety and stress. This increase was found to be motivated by the possible lack of attention to students’ educational needs and failure to achieve academic goals. However, a study by Pressley et al. [51] found that most teachers did not perceive any extra burden during the first period of the pandemic; however, after two months, teachers began to perceive an increase in anxiety, including stress, with teachers who were following virtual instruction experiencing the greatest increase in anxiety. Similarly, a study by Hassan, Mirza and Hussain [52] showed that although many schools are technologically adapted to vicissitudes such as COVID-19, students and teachers are not prepared for its use, either because of poor adaptability or the inability to use these technological devices effectively. This situation can create stress and anxiety for teachers, which hampers their effectiveness as teachers.

Longitudinally, the results of the study by Kareem and Tantia [53] indicated that teachers’ experience and attitudes towards change were positively correlated with resilience and negatively correlated with teacher burnout at the beginning of the pandemic. Throughout the first three months of the pandemic, teachers demonstrated increased burnout and cynicism, but also increased classroom management and a greater sense of accomplishment. In addition, teachers’ cognitive and emotional attitudes towards change became more negative. Similarly, a study by Sokal, Trudel and Babb [54] examined the relationships between teacher stress, teacher self-efficacy, and teacher well-being during the COVID-19 pandemic. The study reported that teachers experienced high levels of stress and low levels of positive feelings such as joy, positivity, happiness and job satisfaction during the COVID-19 pandemic which negatively affected their well-being and self-efficacy. On the other hand, a study by Alea et al. [55] revealed that those teachers with a high level of teaching experience showed a greater capacity to adapt to new methodologies compatible with the COVID-19 situation, showing evidence of less stress and anxiety.

In relation to gender, a study by Dosil Santamaría et al. [56] showed that female teachers show significantly more symptoms of stress and anxiety than men, those with children have more depressive symptoms than those without, and people with chronic pathology or living with others with chronic pathology have more stress, anxiety and depression.

### 1.6. Objective and Hypothesis

The aim of this study is to analyse the effect of the frustration of basic psychological needs, resilience, emotional intelligence and inclusion from the perspective of teachers in time of the COVID-19 pandemic. Therefore, this study aims to analyse how teachers’ emotional intelligence and resilience influence the psychological well-being and educational inclusion of students with special educational needs. To this end, the following hypotheses are proposed: (a) Teachers’ emotional intelligence positively predicts resilience, anxiety, depression and stress. (b) The frustration of basic psychological needs negatively predicts resilience. (c) Resilience will negatively affect anxiety, depression and stress. (d) Anxiety, depression and stress negatively predict educational inclusion.

## 2. Method

### 2.1. Participants

The present study required the participation of 542 teachers of therapeutic pedagogy and special educational needs (258 males and 284 females). The age of the teachers ranged from 33 to 56 years, with a mean of 44.87 (SD = 6.17). The percentage of teachers working in urban areas was 80,99% compared to the 20,01% of teachers that worked in rural areas.

The teachers taught in several schools and educational guidance teams in the provinces of Almería, Granada and Jaén (Spain).

### 2.2. Measurements

**Frustration of psychological needs**. To analyse the frustration of autonomy, competence and social relation needs, the adaptation of the Psychological Need Thwarting Scale (PNTS: [19]) to the Spanish educational context [57] was used. This questionnaire consists of the opening sentence “In my work environment...”, followed by 12 items (4 per factor) aimed at analysing autonomy frustration, competence frustration and social relationship frustration. Teachers had to respond on a Likert scale ranging from 1 (not true at all) to 7 (completely true).

**Resilience**. The Spanish version of the Resilience Scale (RS-14) by Wagnild [58] was used. The RS-14 measures two factors: Factor I: Personal Competence (11 items, self-confidence, independence, decisiveness, resourcefulness and perseverance); Factor II: Acceptance of self and life (3 items, adaptability, balance, flexibility and a stable outlook on life). Teachers were asked to respond on a Likert scale ranging from 1 (strongly disagree) to 7 (strongly agree).

**Emotional Intelligence**. The Trait Meta Mood Scale 24 (TMMS-24) by Fernández-Berrocal, Extremera and Ramos [59]) was used. The scale is composed of 24 items, equally distributed among 3 factors: emotional attention (e.g., I tend to worry a lot about how I feel), emotional clarity (e.g., I almost always know how I feel), and emotional repair (e.g., I try to think positive thoughts even when I feel bad). Teachers were asked to rate their agreement with each item on a 5-point Likert-type scale ranging from 1 (strongly agree) to 5 (strongly disagree).

**Burnout**. The Spanish version [60] of the Maslach Burnout Inventory [41] was used. The scale is composed of 21 items distributed across 3 factors: self-fulfilment, depersonalisation and emotional exhaustion. Participants in the study completed the questionnaire using a Likert scale from 0 (never) to 6 (every day).

**Educational Inclusion**. To measure teachers’ attitudes towards educational inclusion we used Boot and Ainscow’s [61] Index for Inclusion translated and adapted to Spanish by Booth, Simón, Sandoval, Echeita and Muñoz [62]. The questionnaire is composed of a total of 56 items (e.g., “All people who come to this school are welcome”) distributed across 6 sub-factors: A1: building community; A2: establishing inclusive values; B1: developing a school centre for all; B2: organising support for diversity; C1: building a curriculum for all; and C2: orchestrating learning, which in turn is divided into 3 factors: (A) creating inclusive cultures, (B) establishing inclusive policies and (C) developing inclusive practices. Teachers respond using a Likert scale from 0 (disagree) to 3 (agree) with the response options of the original scale.

### 2.3. Procedure

Initially, approval was obtained from the bioethics committee of the University of Almeria in order to begin the present study (Ref. UALBIO 2021/24). Once approval was obtained, the management teams of several educational centres were contacted; we explained in detail the objective of the present study and requested their support. Subsequently, those schools that agreed to participate in the study were contacted, explaining the aim of the study and requesting their participation. Before they could participate in the study, they had to submit a signed informed consent form.

The questionnaires were filled in at the beginning of the department coordination meeting, indicating that the answers would be anonymous and confidential. In addition, a member of the research group was present to answer any questions that might arise. The questionnaires took 25 min to complete.

### 2.4. Data Analysis

The statistical analyses carried out in this study were descriptive statistics: mean, standard deviation and bivariate correlations, as well as reliability analysis, using the SPSS v25 statistical package. Subsequently, a structural equation model (SEM) was carried out to analyse the predictive relationships established in the hypothesised model using the AMOS v20 statistical package.

**Figure 1:**
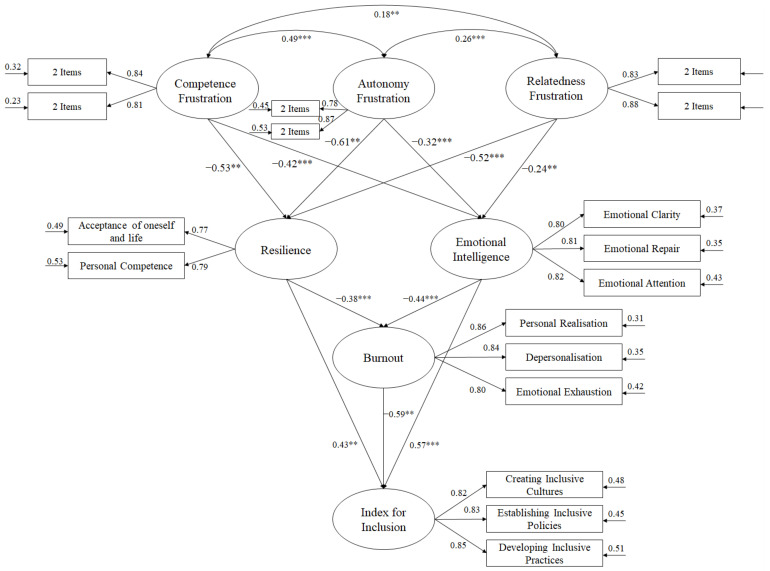
Structural equation model showing the relationships between variables. Note: ** *p* < 0.01; *** *p* < 0.001.

A bootstrapping of 5000 interactions was used to carry out the SEM, together with the maximum likelihood method. To analyse the goodness of fit of the hypothesised model (Figure 1) the following indices were considered [63]: χ2/df, with values between 2 and 3; the Comparative Fit Index (CFI), Incremental Fit Index (IFI), and Tucker–Lewis Index (TLI), with values above 0.95; the Root Mean Square Error of Approximation (RMSEA) plus its 90% confidence interval with values below 0.06; and the Standardized Root Mean Square Residual (SRMR) with values below 0.08. Nevertheless, these indices should be interpreted with caution as they can be restrictive when the model is very complex [63].

## 3. Results

### 3.1. Preliminary Analysis

Table 1 shows the mean, standard deviation, and bivariate correlations. The correlations reflected a positive relationship between the factors of frustration with each other and with burnout. Similarly, positive correlations were also reflected between emotional intelligence, resilience and the inclusion index. In addition, Table 1 shows the reliability analyses with all scores being above 0.80 [64].

### 3.2. Structural Equation Modelling

Testing the hypothesised predictive relationship model on teachers (Figure 1) revealed the following fit indices: χ2 (105. N = 542) = 301.63, *p* < 0.001; χ2/df = 2.87; CFI = 0.96; IFI = 0.96; TLI = 0.96 RMSEA = 0.054 (90% CI = 0.050–0.061); and SRMR = 0.039.

The relationships obtained between the different factors that made up the model were as follows:

(a) The correlations between each of the basic psychological need frustrations were positive: β = 0.49 (*p* < 0.01) between competence and autonomy frustration; β = 0.26 (*p* < 0.01) between autonomy and relatedness frustration with others; and β = 0.18 (*p* < 0.01) between competence and relatedness frustration;

(b) The relationship between competence frustration, resilience (β = −0.53, *p* < 0.01) and emotional intelligence (β = −0.42, *p* < 0.01) was negative;

(c) The relationship between autonomy frustration, resilience (β = −0.61, *p* < 0.01) and emotional intelligence (β = −0.32, *p* < 0.001) was negative;

(d) The relationship between relatedness frustration, resilience (β = −0.52, *p* < 0.001) and emotional intelligence (β = −0.24, *p* < 0.01) was negative;

(e) The relationship between resilience and burnout (β = −0.38, *p* < 0.001) was negative, whereas with the inclusion index (β = 0.43, *p* < 0.01) was positive;

(f) The relationship between emotional intelligence and burnout (β = −0.44, *p* < 0.001) was negative, whereas with the inclusion index (β = 0.57, *p* < 0.001) was positive;

(g) The relationship between burnout and the inclusion index (β = −0.59, *p* < 0.01) was negative.

## 4. Discussion

Teaching is one of the most stressful work activities, as the workload inherent to the activity has an effect on teachers’ relationships with colleagues and students. This involves a great deal of psychological and emotional stress that is sometimes difficult to cope with without a series of internal mechanisms that allow the teacher to adapt to these difficulties. In addition to this situation, in recent years, COVID-19 has led to an excessive workload and an increase in fear, stress and anxiety given the hygienic measures to be implemented and the modifications in teaching methodologies. Therefore, the study aimed to analyse the psychological coping mechanisms of teachers when facing burnout symptoms arising from daily challenges, and the consequences this has on their inclusive behaviours. For this purpose, a SEM was performed in order to analyse the predictive relationships between the study variables whose values were in accordance with the pre-military analyses with bivariate correlations. In addition, reliability analyses showed a score above 0.70, implying that the variables were related to the unobservable magnitude of interest.

In relation to the main analysis used in this study through SEM, the results obtained show that BPN frustration was negatively related to resilience and emotional intelligence. These results are difficult to compare with previous studies involving teachers, especially if all variables are taken into account at the same time. However, these results are similar to previous studies involving students and athletes. In this regard, a study by Trigueros et al. [65] showed that NPB frustration negatively predicted athletes’ ability to adapt to the demands and challenges faced during competitions or training. Similarly, a study by Waterschoot et al. [66] with university students showed that BPN frustration reduced students’ attention during lectures due to a decrease in students’ sense of adaptation to difficulties. On the other hand, a study by Lera and Tawahina [67] with 300 adolescents from conflict zones showed that the traumatic experiences of these young people were negatively related to contextual resilience. On the other hand, a study by van der Kaap-Deeder et al. [68] with university students showed that BPN frustration negatively influenced emotion management and regulation, factors linked to emotional intelligence. On the other hand, a study by Abidin et al. [69] with parents showed that BPN satisfaction was positively related to emotional well-being, while BPN frustration was negatively related. On the other hand, a study by Trigueros-Ramos et al. [70] with secondary school students showed that teacher autonomy support increases students’ enjoyment, motivation and confidence in sport, i.e., if the teacher had EI, he/she could satisfy his/her NPBs and thus students’ educational inclusion.

On the other hand, resilience and EI have been negatively related to burnout. However, these results cannot be contrasted with studies where each of the variables are grouped together, although they can be contrasted separately. In this sense, a study by Howard and Johnson [71] with primary school teachers showed that high levels of high resilience were associated with low levels of burnout and job stress. Similarly, a study by Richards et al. [72] analysed the impact of resilience on stress and burnout in primary and secondary school teachers. This study showed that those teachers who were highly adaptive and fluent in the use of adaptive strategies had low levels of job stress and burnout. A study by Polat and İskender [73] analysed the relationship between resilience and teachers’ job satisfaction through burnout. The results showed that high resilience was related to low levels of burnout. Regarding EI and burnout, a study by Lee and Chelladurai [74] with university teachers showed that high levels of emotional intelligence were related to low levels of burnout. Similarly, a study by Zysberg et al. [75] showed that high levels of emotional intelligence were related to low levels of stress and burnout, especially in the workplace. In this line, it can be affirmed that teachers who have a high level of EI and resilience are less affected by the effects of burnout, which implies less chronic fatigue, demotivation and a decrease in job dissatisfaction, acting as a protective barrier against the physical and emotional exhaustion that burnout produces. In this sense, resilience can be an empowering instrument to work on in formal education [76] as a tool to resolve difficult situations that teachers face in the professional and personal spheres. As for EI, this intelligence plays an important role in self-esteem and self-confidence [77], helping to overcome life’s difficulties, as well as to develop greater resilience. In other words, the positive correlation between EI and resilience is also associated with greater life satisfaction and in this case, this is corroborated by the study in older adults by Meléndez et al. [78] who found in their research that the dimensions of emotional clarity and emotional regulation were significant and positive predictors of resilience.

Finally, the results showed that emotional intelligence and resilience were positively related to educational inclusion, while burnout was negatively related. These results have been found to be partially similar with previous studies, although not with all variables. In this regard, a study by Fabio and Palazzeschi [79] showed that teacher effectiveness and teacher engagement during lessons was influenced by high levels of resilience and emotional intelligence. Therefore, emotional intelligence could increase teachers’ self-efficacy beliefs and resilience. Moreover, this effect could be inverse, i.e., teachers’ self-efficacy and resilience could increase their emotional intelligence by improving the ability to recognise and manage one’s own emotions in stressful situations [80].

As limitations of the study, it could be highlighted that there is a lot of research on BPN satisfaction and very little on BPN frustration, considering that the frustration of autonomy, competence and relationships with others usually leads to more problems for teachers in the performance of their work, raising the risk of suffering burnout implicitly. Moreover, this is a relational study that does not allow the establishment of causal relationships; thus, the results obtained can be interpreted in many ways, given that the study was carried out by means of a self-administered questionnaire. Future research should investigate the reasons for the frustration of NPBs and subsequent burnout, as well as identify what motivates teachers to develop resilience and thus inclusive educational practices. In addition, future studies could investigate the differences between teachers in urban and rural schools.

## 5. Conclusions

The present study has shown the protective factor of resilience and emotional intelligence against burnout, leading to more inclusive behaviour in the classroom. However, the high stress of the teaching job and unforeseen situations such as the COVID-19 pandemic make teachers vulnerable to stress, depression, anxiety and high physical and emotional demands. This leads to a decline in the quality of teaching and care for students with SEN. Therefore, education authorities should allocate reasonable resources to identify and support school teachers by providing them with the necessary tools and resources [81]. In addition, public administrations should provide teachers with educational practices on ICT tools to help them cope with future and present situations that make physical teaching difficult so that confidence is strengthened, choice is maximised, and teacher empowerment is prioritized [82].

## Figures and Tables

**Table 1:** Mean, standard deviation, internal consistency analysis and bivariate correlations.

Factors	*M*	*SD*	α	1	2	3	4	5	6	7
1. Frustration Competence	2.03	1.26	0.81	-	0.42 ***	0.49 ***	−0.31 ***	−0.55 ***	0.29 ***	−0.54 ***
2. Frustration Autonomy	2.19	1.47	0.82		-	0.37 ***	−0.47 ***	−0.54 ***	0.34 **	−0.41 **
3. Frustration Relatedness	2.21	1.24	0.80			-	−0.58 ***	−0.75 ***	0.21 **	−0.21 **
4. Emotional Intelligence	3.46	1.19	0.84				-	0.64 ***	−0.37 ***	0.67 ***
5. Resilience	5.28	1.20	0.85					-	−0.71 ***	0.62 ***
6. Burnout	1.39	1.10	0.80						-	−0.48 **
7. Inclusion Index	2.10	0.59	0.82							-

** *p* < 0.01; *** *p* < 0.001. Note: α= Cronbach’s alpha.

## Data Availability

Not applicable.
